# Therapeutic Notes

**Published:** 1898-02-19

**Authors:** 


### THERAPEUTIC NOTES.

### Combined Remedies in Heart Diseases.

Dr. Seymour Taylor says that remedies given together often act well, although given separately they may fail to act at all. "I can remember distinctly two patients this year who were apparently dying, and who had had digitalis, strophanthus, and convallaria at different times, but not together. We gave them digitalis, convallaria, and strophanthus, and in both cases the men were sitting up in bed or in a chair with limbs almost quite free from dropsy in a week."

